# Effect of end-of-production light treatment under different light/dark alternating frequencies on ascorbic acid accumulation and metabolism of hydroponic lettuce

**DOI:** 10.3389/fpls.2025.1681893

**Published:** 2025-12-03

**Authors:** Dong Yu, Zonggeng Li, Jiangtao Hu, Fang Wang, Wei Lu, Quan Yuan, Sen Wang, Chengbo Zhou, Qichang Yang

**Affiliations:** 1 Institute of Urban Agriculture, Chinese Academy of Agricultural Sciences, Chengdu, China; 2 College of Horticulture, Sichuan Agricultural University, Chengdu, China

**Keywords:** ascorbic acid (AsA), light/dark alternating, end-of-production (EOP), reactive oxygen species (ROS), hydroponic lettuce

## Abstract

**Introduction:**

This study investigated the effects of different light/dark alternation cycles on ascorbic acid (AsA) metabolism and oxidative stress in hydroponic lettuce under end-of-production (EOP) conditions characterized by high light intensity and a high blue-light ratio (500 mmol·m⁻²·s⁻¹, red:blue = 1:1).

**Methods:**

Four treatments were applied: RB1 (continuous illumination), RB2 (4 h light/4 h dark/4 h light), RB4 (three cycles of 2 h light/2 h dark plus a final 2 h light), and RB8 (seven cycles of 1 h light/1 h dark with a final 1 h light).

**Results:**

The results revealed that AsA content exhibited an initial increase followed by a decrease with increasing alternation frequency, peaking under the RB2 treatment. Specifically, RB2 achieved the highest AsA accumulation, which was 11.8%, 28.5%, and 41.6% greater than in RB1, RB4, and RB8, respectively. This enhancement was attributed to the synchronous upregulation of the gene expression and enzymatic activity of GLDH, as well as key enzymes in the ascorbate-glutathione cycle (APX, MDHAR, and DHAR). Oxidative stress indicators (H₂O₂, MDA) decreased with increased light/dark alternation frequency.

**Discussion:**

Therefore, the RB2 treatment, by coordinating reactive oxygen species (ROS) induction and promoting the simultaneous upregulation of expression and activity in the AsA metabolic enzyme system, serves as an energy-efficient strategy for improving the nutritional quality of lettuce in controlled environments.

## 1 Introduction

Ascorbic acid (AsA), also referred to as reduced ascorbate, is essential for photosynthesis, cell proliferation, signal transduction, and defense against oxidative stress (Smirnoff, 2000; Smith et al., 2007; Ma et al., 2014; Ntagkas et al., 2019). In higher plants, AsA is mainly synthesized via the D-mannose/L-galactose pathway, where key enzymes such as GDP-mannose-3,5-epimerase (GME), GDP-L-galactose phosphorylase (GGP), L-galactose-1-phosphate phosphatase (GPP), and L-galactono-1,4-lactone dehydrogenase (GLDH) catalyze sequential steps (Wheeler et al., 1998; Smirnoff, 2018). Its redox homeostasis is maintained through recycling, in which ascorbate peroxidase (APX), monodehydroascorbate reductase (MDHAR), dehydroascorbate reductase (DHAR), and glutathione reductase (GR) play central roles (Apel and Hirt, 2004; Ntagkas et al., 2018; Zha et al., 2020). Therefore, assessing the activities and expression levels of these enzymes provides an effective means to evaluate AsA biosynthesis and accumulation under varying light conditions.

Light is a key regulator of AsA metabolism in higher plants, modulating both gene expression and enzyme activities that determine the AsA pool size (Zha et al., 2019a). Elevated light intensities generally promote AsA accumulation (Bartoli et al., 2006; Ntagkas et al., 2019). Our previous study showed that end-of-production (EOP) high light irradiation (500 μmol·m^-^²·s^-1^, 8 h) significantly enhanced AsA and total ascorbate (T-AsA) contents in lettuce (Zhou et al., 2021). Thus, EOP high light represents a cost-effective pre-harvest strategy to improve quality while saving energy (Gómez and Jiménez, 2020; Min et al., 2021). Beyond light intensity, the spectral composition of light also decisively influences crop nutritional quality (Lin et al., 2013). Red and blue spectra, perceived by photoreceptors such as phytochromes, phototropins, and cryptochromes, are critically involved in regulating plant development, carbohydrate storage, and secondary metabolite synthesis (Landi et al., 2020; Wollaeger and Runkle, 2015). Notably, blue light has been shown to effectively enhance AsA biosynthesis in leaves of multiple species, including *Lactuca sativa*, *Spinacea oleracea*, and *Avena sativa* (Zha et al., 2020; Ohashi-Kaneko et al., 2007; Mastropasqua et al., 2012). Consistent with this, our previous study demonstrated that under EOP high light (500 μmol·m^-^²·s^-1^), red: blue light ratios of 1:1 and 2:1 resulted in higher AsA content in lettuce than did ratios of 3:1 and 4:1 (Zhou et al., 2023). However, while high light intensity and a high blue light fraction are effective in stimulating AsA synthesis, they also readily induce photooxidative stress, leading to substantial accumulation of reactive oxygen species (ROS) (Zhou et al., 2021, 2023; Chibani et al., 2025). Therefore, rhythmic strategies like EOP light/dark alternation, which coordinate photosynthesis with redox balance, represent a promising avenue for using EOP high light and high blue light to enhance quality. This approach warrants further research for its practical applications.

Previous studies have demonstrated that short-term dark periods can regulate plant oxidative stress. Exposure to short-term darkness significantly reduced ROS levels in geranium (Rosenwasser et al., 2010) and decreased MDA content in *Arabidopsis* suspension cells compared with those under light exposure (Wang et al., 2012), both effects being associated with alleviated lipid peroxidation due to the cessation of photosynthesis. However, these studies have primarily focused on continuous darkness, leaving the effects of light/dark alternations insufficiently explored. Moreover, the light/dark cycle has been confirmed as a key factor regulating plant metabolism. For instance, it governs anthocyanin biosynthesis in strawberries (Kurata et al., 2000) and enhances both biomass and carbohydrate content in lettuce (Chen and Yang, 2018). This collective evidence suggests that integrating the stress-mitigating effect of darkness with the growth-regulatory role of light/dark cycles could simultaneously alleviate oxidative stress and promote nutrient accumulation in lettuce.

In lettuce, a common leafy vegetable, AsA serves as a key indicator of nutritional quality. Our previous studies have confirmed that high light combined with a high proportion of blue light during the EOP stage effectively promotes AsA accumulation, but it also induces photooxidative stress. Therefore, this study introduced light/dark alternation as an additional variable under EOP conditions of high light intensity and a high blue light ratio (500 μmol·m^-^²·s^-1^, red: blue = 1:1). This study was designed to evaluate the efficacy of this approach in alleviating oxidative stress and to elucidate the regulatory mechanisms governing AsA metabolism. All treatments were supplied with the same total daily light integral (8 h) but were applied at different light/dark alternation frequencies (RB1, RB2, RB4, RB8). Samples were immediately collected after the final light period to measure plant growth parameters, soluble sugar content, as well as the activity and gene expression of key enzymes in the GLDH and AsA cycles (e.g., APX, MDHAR). The specific objectives were: (1) to elucidate the mechanisms of AsA metabolism and accumulation in lettuce leaves under varying light/dark alternation frequencies; and (2) to identify the optimal alternation frequency that concurrently alleviates oxidative stress and promotes AsA synthesis, thereby improving lettuce nutritional quality and providing a theoretical basis for optimized light management in protected cultivation.

## 2 Materials and methods

Lettuce (*Lactuca sativa* L.) seeds were sown in pre-hydrated sponge blocks (2.5 cm^3^) within propagation trays. After 48 h of imbibition, seeds were exposed to white LED light (200 μmol·m^-^²·s^-1^; 16 h photoperiod from 06:00 to 22:00) until two true leaves developed. Uniform seedlings were selected, rinsed three times with deionized water, and transplanted into hydroponic tanks with recirculating nutrient solution. Plants were cultivated for 16 days under red-blue LEDs (200 μmol·m^-^²·s^-1^; red: blue ratio = 3:1) in a controlled environment (23 ± 3°C, 65 ± 5% RH, 420 ± 5 ppm CO_2_). The modified Hoagland solution contained (mM): 4 Ca(NO_3_)_2_·4H_2_O, 6 KNO_3_, 1 NH_4_H_2_PO_4_, 2 MgSO_4_·7H_2_O; (μM): 71 Fe-EDTA-Na_2_, 46 H_3_BO_3_, 9.6 MnSO_4_·4H_2_O, 0.8 CuSO_4_·5H_2_O, 0.07 (NH_4_)_6_Mo_7_O_24_·4H_2_O (EC≈1.63 mS·cm^-1^, pH≈5.7), with weekly replacement of the nutrient solution.

**Figure 1:**
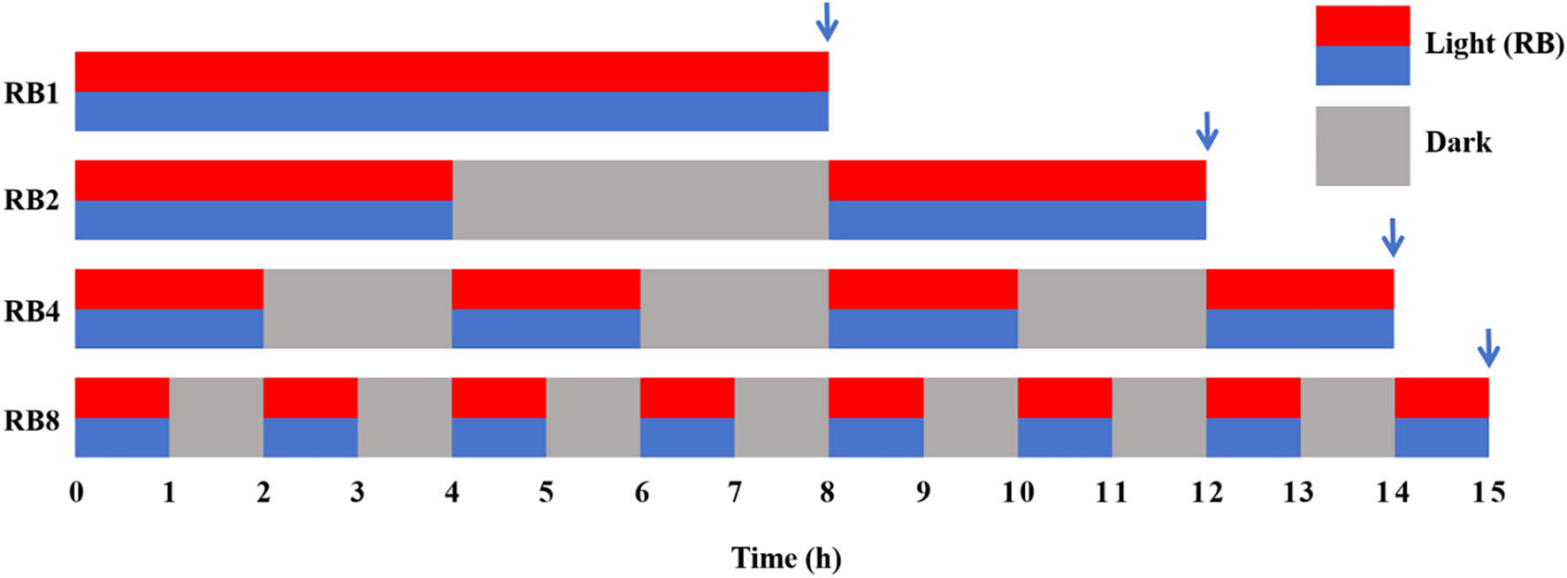
Experimental design of light treatment with different light/dark alternation frequencies at the end-of-production (EOP). The ratio of red to blue light intensity was 1R:1B. The arrow points to the sampling time of each treatment.

After 16 days of hydroponic cultivation, when the lettuce plants had developed approximately 16–18 fully expanded leaves (**Supplementary Figure S1**), four photoperiod treatments were initiated at 06:00 on day 17. For each treatment, 27 uniformly growing plants were randomly selected. Additionally, eight uniformly growing plants (independent of the 27 treatment plants) were used for baseline measurements (T0): four were randomly selected for growth measurements before treatment, and the entire set of leaves (the 1st to 5th fully expanded leaves counted from the top of the plant downward, with petioles removed) was collected from the other four plants. These leaf samples were immediately flash-frozen in liquid nitrogen and stored at −80°C for subsequent analyses. All treatments delivered a total of 8 h of high light exposure (500 μmol·m^-^²·s^-1^; red: blue = 1:1) through specific intermittent protocols (**Figure 1**): RB1 (continuous illumination), RB2 (4 h light followed by 4 h dark and concluding with a final 4 h light), RB4 (three cycles of 2 h light/2 h dark plus a final 2 h light), and RB8 (seven cycles of 1 h light/1 h dark with a final 1 h light). A custom LED lighting system (Wuxi Huazhaohong Optoelectronic Technology Co., Ltd., China) with spectral peaks at 662 nm (red) and 460 nm (blue) was positioned 30 cm above the plant canopy. The photosynthetic photon flux density (PPFD) at the canopy level was verified using a calibrated spectrometer (Avaspec-ULS2048, Avantes, Netherlands). Following the final light period, sampling was conducted for the treatment groups. Leaf tissue was collected using the same protocol as for the T0 biochemical samples, with each plant considered an independent biological replicate. All collected tissues were ground in liquid nitrogen and stored at −80°C until analysis.

### 2.1 Growth measurement

Four representative lettuce plants per treatment were harvested by cutting at the crown. Shoot and root fresh weights were immediately measured using an analytical balance. All leaves were detached for morphological analysis: total leaf count was recorded, and leaf area quantified with a calibrated leaf area meter (LI-3100C, LI-COR, Lincoln, NE, USA). Plant tissues were oven-dried in paper bags at 80°C for 72 h until constant weight, with shoot and root dry weights determined using an analytical balance.

### 2.2 Determination of soluble sugar content

The soluble sugar content in lettuce leaves was determined using a plant soluble sugar content assay kit (Beijing Solarbio Science & Technology Co., Ltd.; Product No. BC0030; the kit adopts the anthrone colorimetric method, which is applicable for the determination of soluble monosaccharides, oligosaccharides and polysaccharides in lettuce leaf samples; Website: https://www.solarbio.com). Accurately weighed frozen leaf tissue (0.1 g) was transferred into pre-chilled 2 mL centrifuge tubes. Two 3 mm diameter stainless steel grinding beads were then added, and the samples were homogenized using a cryogenic grinder (Scientz-48, Ningbo Scientz Biotechnology Co., Ltd., China) under liquid nitrogen. Subsequently, 1 mL deionized water was added, vortexed thoroughly, and extracted in a boiling water bath for 10 min. After cooling to room temperature, the homogenate was centrifuged at 8,000 × g for 10 min. The supernatant was analyzed following the kit protocol using a spectrophotometer (Shimadzu UV-1800, Kyoto, Japan) at 620 nm.

### 2.3 AsA and T-AsA content determination

The contents of AsA and T-AsA in lettuce leaves were quantified using ultra-high-performance liquid chromatography (UPLC, H-Class, Waters Corporation, USA) with an HSS T3 column (2.1 mm × 100 mm, 1.8 μm; Waters) and a photodiode array detector (PDA), as previously described (Shiroma et al., 2019; Spínola et al., 2012). Frozen leaf tissue (0.1 g) was homogenized as described above. Subsequently, 1 mL of ice-cold extraction buffer containing 1.5% (w/v) metaphosphoric acid, 4% (w/v) glacial acetic acid, and 0.5 mM EDTA was added. The mixture was vortexed, sonicated for 10 min in an ice bath, then centrifuged at 15,000 × g (4°C, 20 min). A 50 μL aliquot of supernatant was mixed with 190 μL of 200 mM Tris-HCl buffer, 10 μL of ultrapure water, and 50 μL of 0.4 M H_2_SO_4_. After 30 min of incubation at 25°C, samples were filtered through 0.22 μm PTFE membranes (Jinteng Experimental Equipment Co., Ltd., China) and analyzed at 245 nm. L-ascorbic acid was used as the standard solution with concentrations ranging from 0.05 to 2 μg·mL^-1^, and the calibration curve showed good linearity (*R*² = 0.999). The mobile phase comprised 0.1% (v/v) formic acid at a flow rate of 250 μL·min^-1^ with an isocratic elution mode. For T-AsA determination, the reduced ascorbate pool was measured by replacing the 10 μL of ultrapure water in the derivatization step with 10 μL of 750 mM dithiothreitol (DTT) to reduce dehydroascorbate (DHA) to AsA.

### 2.4 Extraction and activity determination of enzymes

GLDH activity was determined using a commercial assay kit (Beijing Solarbio Science & Technology Co., Ltd.; Product No. BC1250; the kit is based on the principle that GLDH catalyzes the reduction of oxidized cytochrome c (Cytc) by L-galactonolactone. Reduced Cytc has a characteristic absorption peak at 550 nm, and GLDH activity is calculated by measuring the rate of increase in reduced Cytc; Website: https://www.solarbio.com) following the manufacturer’s protocol. Ascorbate peroxidase (APX) activity was analyzed following the method of Zha et al. (2019a). Frozen leaf tissue (0.3 g) was homogenized as described above. The homogenate was mixed with 2 mL of ice-cold extraction buffer [100 mM potassium phosphate (pH 7.0), 0.2 mM EDTA, 1 mM ascorbic acid (AsA)] and sonicated for 10 min in an ice bath. After centrifugation at 15,000 × g (4°C, 10 min), 0.1 mL of supernatant was reacted with 2.9 mL of reaction mixture containing 50 mM potassium phosphate (pH 7.8), 0.5 mM AsA, and 0.25 mM H_2_O_2_. The reaction was initiated by adding the enzyme extract, and the absorbance of the reaction mixture was measured at 290 nm. One unit of enzyme was defined as the amount that oxidized 1 μmol of AsA per min. The activities of dehydroascorbate reductase (DHAR), monodehydroascorbate reductase (MDHAR), and glutathione reductase (GR) were assayed according to Ma and Cheng (2004), with minor modifications. Frozen leaf tissue (0.1 g) was homogenized as described above. After grinding, 1 mL ice-cold extraction buffer [50 mM potassium phosphate buffer (pH 7.5), 1 mM DTT, 0.1% (v/v) Triton X-100, 0.2% (v/v) β-mercaptoethanol, 2% (w/v) polyvinylpyrrolidone, 1 mM EDTA] was added, followed by 10 min ice-bath sonication. The homogenate was centrifuged at 15,000 × g (4°C, 20 min). DHAR activity: 0.1 mL of supernatant was mixed with 2.9 mL of reaction mixture containing 100 mM HEPES-KOH (pH 7.0), 2.5 mM glutathione (GSH), 1 mM EDTA, and 0.6 mM dehydroascorbic acid (DHA). The reaction was initiated by adding the enzyme extract, and absorbance was recorded at 265 nm. One unit of the enzyme was defined as the amount that reduced 1 μmol of DHA per min. MDHAR activity: 0.1 mL of supernatant was combined with 2.9 mL of reaction mixture [50 mM HEPES-KOH (pH 7.6), 0.3 mM NADH, 0.5 mM AsA, 0.5 U ascorbate oxidase (AO)]. The reaction was initiated by adding AO, and absorbance was monitored at 340 nm. One unit of the MDHAR was defined as the amount that oxidized 1 μmol of NADH per min. GR activity: 0.1 mL of supernatant was reacted with 2.9 mL of reaction mixture [100 mM Tris-HCl (pH 8.0), 1 mM EDTA, 3 mM oxidized glutathione (GSSG), 0.6 mM NADPH]. The reaction was started by adding NADPH, and absorbance was measured at 340 nm. One unit of the enzyme was defined as the amount that oxidized 1 μmol of NADPH per min.

### 2.5 Hydrogen peroxide and superoxide anion content were determined

H_2_O_2_ content was determined using a commercial kit (Comin Biotechnology Co., Ltd., Suzhou, China; Product No. H2H2-2-Y; the kit is based on the principle that H_2_O_2_ reacts with titanium sulfate to form a yellow titanium peroxide complex which has a characteristic absorption at 415 nm; Website: https://www.cominbio.com), and the measurement was performed according to the manufacturer’s instructions.

The O_2_^-^ generation rate was determined using a commercial assay kit (Beijing Solarbio Science & Technology Co., Ltd., China; Product No. BC1415; Website: https://www.solarbio.com) based on the sulfamate colorimetric method described by Elstner and Heupel (1976). Frozen leaf tissue (0.1 g) was homogenized as described above. 1 mL of 50 mM phosphate buffer (pH 7.8) [1 mM EDTA, 0.3% (v/v) Triton X-100, 2% (w/v) polyvinylpyrrolidone] was added to the centrifuge tube. After centrifugation at 10,000 × g (4°C, 20 min), 0.5 mL of supernatant was mixed with 0.4 mL of 50 mM hydroxylamine hydrochloride and incubated at 37°C for 20 min. Subsequently, 0.3 mL of 17 mM sulfanilic acid and 0.3 mL of 7 mM α-naphthylamine were added sequentially, followed by a 37°C incubation for 20 min. After adding 0.5 mL of chloroform, the mixture was centrifuged at 8,000 × g (25°C, 5 min). Finally, 1 mL of the upper aqueous phase was collected and its absorbance was measured at 530 nm.

### 2.6 Determination of malondialdehyde content

The MDA content in plant tissues was determined according to the method of Hodges et al. (1999), which is based on Heath and Packer (1968), with modifications. Frozen leaf tissue (0.1 g) was homogenized as described above. After adding 1 mL of ice-cold 10% (w/v) trichloroacetic acid (TCA), the homogenate was centrifuged at 8,000 × g (4°C, 10 min). Subsequently, 0.5 mL of supernatant was mixed with 0.5 mL of 0.6% (w/v) thiobarbituric acid (TBA) dissolved in 10% TCA and heated at 100°C for 30 min in a water bath, then cooled to room temperature and centrifuged at 10,000 × g (25°C, 10 min). A 200 μL aliquot of the supernatant was transferred to a 96-well plate, and absorbance was measured at 450, 532, and 600 nm.

### 2.7 RNA extraction and expression determination

Total RNA was extracted from lettuce leaves using an RNAprep Pure Plant Plus Kit (Tiangen Biotech Co., Ltd., Beijing, China; Product No. DP441; Website: https://www.tiangen.com), and RNA quality and concentration were assessed with an ultramicro spectrophotometer (TECAN, Infinite M200 Pro, Switzerland). Reverse transcription was performed with the Fast King RT kit (KR116, Tiangen), and RT-qPCR was carried out on a CFX96 system (Bio-Rad, USA) using diluted cDNA and TransStart Top Green qPCR SuperMix (TransGen, Beijing, China). The program was 95°C for 3 min, followed by 40 cycles of 95°C for 10 s, 60°C for 15 s, and 72°C for 15 s. *18S rRNA* served as the internal control gene, and gene expression levels were normalized to T0 (set to 1) and calculated using the 2^–(ΔΔCt)^ method (Livak and Schmittgen, 2001). The heatmap depicting gene expression was plotted using TBtools-II (https://github.com/CJ-Chen/TBtools-II). The fold-change values relative to T0 were log_2_-transformed, with down-regulation shown in blue, no change at 0 as the midpoint, and up-regulation in red. Each treatment included four biological replicates, each with three technical replicates. Gene-specific primers were designed with NCBI Primer-BLAST, synthesized by Sangon Biotech (Shanghai, China), and are listed in **Table 1**.

**Table 1:** Nucleotide sequences of specific primers used for RT-qPCR.

Gene	Forward primer (5’-3’)	Reverse primer (5’-3’)	Tm (°C)	Product length (bp)
*18S rRNA*	AAGCCCGATCCAGCAATAT	GGCGACTTTCACTTTCAACC	54.06	213
*GLDH*	GAAGCAGAAGATCCGTCCTG	TACCTGAACAATGCCACCAA	54.83	244
*APX*	TTCTATCAGTTGGCTGGTGTTG	TACTTGCCTCAAATGGTCGTT	54.39	147
*MDHAR1*	AGTTGGAGGTGGAAATGCTG	ACCTCCTGAACCCACACAAG	56.34	199
*MDHAR2*	TGAAGTTGTATGGTGACATCAGAAG	AGATCGAAGGAACGCGAGTAG	55.44	154
*DHAR1*	CTTGCCGAGAAGGGTGTTT	TTTGCCGTTGTGGATGAGA	54.87	120
*DHAR2*	TGAGGTCTGTTGCAAAGCTG	CAACGGAACTTTCCCATTTG	53.74	178
*GR*	AGGACGAGGAAAGATTGTGGA	GCAAACTCAAGGGCAATGTAA	55.33	213

### 2.8 Data statistics and analysis

Statistical analysis was performed using SPSS 18.0 (IBM SPSS Statistics, Chicago, IL, USA). One-way ANOVA followed by Duncan’s multiple range test was conducted for multiple comparisons, with statistical significance set at *p* < 0.05.

## 3 Results and analysis

### 3.1 Effect of EOP light treatment under different light/dark alternating frequencies on hydroponic lettuce growth parameters

The treatments of light/dark alternation at different frequencies had no significant impact on the growth parameters of hydroponic lettuce before harvest (**Table 2**). There were no significant differences in indicators such as leaf area, shoot fresh/dry weight, root fresh/dry weight among the treatments. The results indicate that changes in the frequency of light/dark alternation applied did not significantly affect the overall growth trend of lettuce.

**Table 2:** The leaf area, shoot fresh weight, shoot dry weight, root fresh weight, and root dry weight of lettuce plants grown under different light treatments at the end-of-production (EOP) stage: RB1 (continuous illumination), RB2 (4 h light followed by 4 h dark and concluding with a final 4 h light), RB4 (three cycles of 2 h light/2 h dark plus a final 2 h light), and RB8 (seven cycles of 1 h light/1 h dark with a final 1 h light).

Treatment	Leaf area (cm^2^)	Shoot fresh weight (g)	Shoot dry weight (g)	Root fresh weight (g)	Root dry weight (g)
RB1	841.72±74.55 a	47.72±7.04 a	2.13±0.10 a	7.03±0.28 a	0.260±0.05 a
RB2	920.41±90.03 a	53.61±3.65 a	2.15±0.19 a	7.30±0.68 a	0.259±0.05 a
RB4	919.87±59.02 a	52.59±6.78 a	2.13±0.16 a	7.67±0.31 a	0.284±0.02 a
RB8	861.68±68.53 a	49.37±8.27 a	2.01±0.37 a	6.88±0.27 a	0.248±0.01 a

Values represent the means of four replicates ± SD. Different letters indicate significant differences using Duncan’s multiple range test (*p* < 0.05; n = 4).

### 3.2 Effect of EOP light treatment under different light/dark alternating frequencies on AsA pool

After 8 h light treatment exposure with different light/dark cycle frequencies. The RB2 treatment induced the highest AsA accumulation (426.9 μg·g^-1^ FW), with increases of 11.8%, 28.5%, and 41.6% compared to the RB1, RB4, and RB8 treatments, respectively. Similarly, RB2 achieved maximum T-AsA content, exceeding RB1, RB4, and RB8 by 7.6%, 16.4%, and 25.7%, respectively (**Table 3**).

**Table 3:** AsA (A) and T-AsA (B) contents in lettuce leaves of plants grown under different light treatments at the end-of-production (EOP) stage: T0 (pre-treatment baseline), RB1 (continuous illumination), RB2 (4 h light followed by 4 h dark and concluding with a final 4 h light), RB4 (three cycles of 2 h light/2 h dark plus a final 2 h light), and RB8 (seven cycles of 1 h light/1 h dark with a final 1 h light).

Treatment	AsA (μg·g^-1^ FW)	T-AsA (μg·g^-1^ FW)
T0	203.64±23.62 d	291.22±21.09 d
RB1	381.87±31.52 b	503.66±34.20 ab
RB2	426.92±27.71 a	541.83±43.43 a
RB4	332.12±20.41 c	465.63±28.97 bc
RB8	301.50±18.97 c	430.99±17.76 c

Values represent the means of four replicates ± SD. Different letters indicate significant differences using Duncan’s multiple range test (*p* < 0.05; n = 4).

### 3.3 Effect of EOP light treatment under different light/dark alternating frequencies on *GLDH* expression and activity in hydroponic lettuce

**Figure 2:**
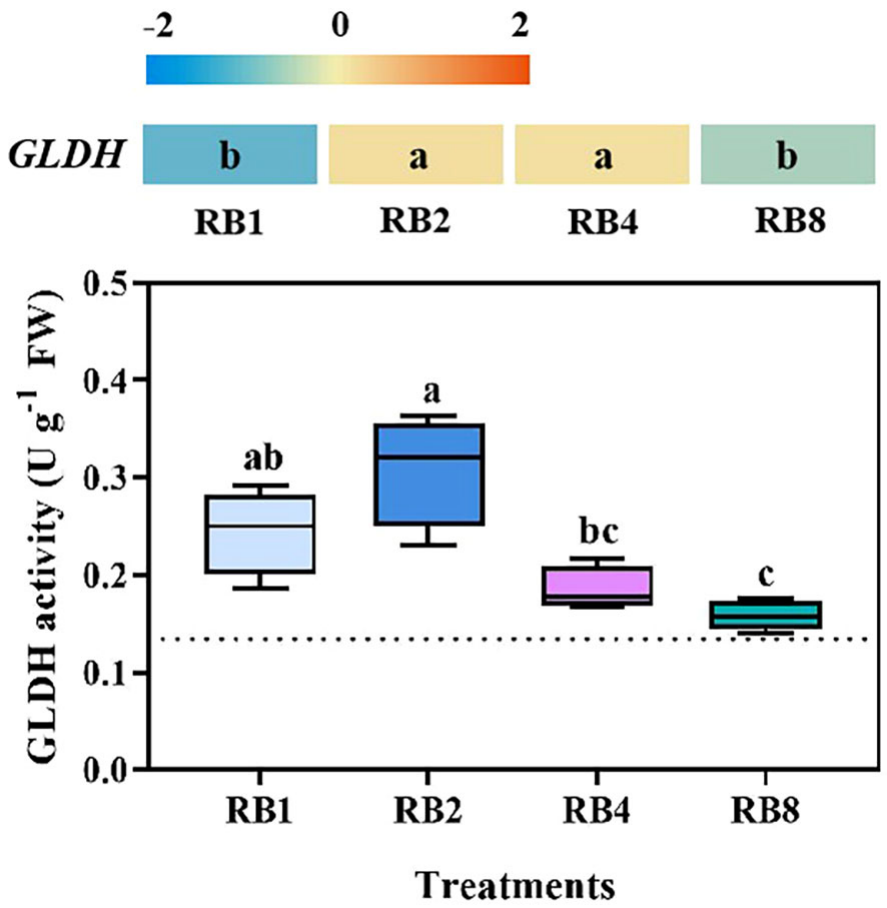
*GLDH* expression and GLDH activity in lettuce leaves of plants grown under different light treatments at the end-of-production (EOP) stage: RB1 (continuous illumination), RB2 (4 h light followed by 4 h dark and concluding with a final 4 h light), RB4 (three cycles of 2 h light/2 h dark plus a final 2 h light), and RB8 (seven cycles of 1 h light/1 h dark with a final 1 h light). The dotted line indicates the sampling before EOP irradiation. Different letters indicate significant differences using Duncan’s multiple range test (*p* < 0.05; n = 4). Gene expression analyses were performed with four biological replicates, each including three technical replicates.

EOP light/dark alternation differentially regulated *GLDH* in lettuce leaves (**Figure 2**). RT-qPCR analysis revealed that *GLDH* transcript levels were significantly upregulated under RB2 and RB4 treatments compared to RB1 and RB8, with the lowest transcriptional levels observed in the RB1 treatment group (**Supplementary Table S4**). Enzymatic assays revealed the highest GLDH activity in RB2-treated leaves, being significantly greater than in RB4 and RB8, but statistically comparable to RB1.

### 3.4 Effect of EOP light treatment under different light/dark alternating frequencies on AsA cycle-related gene expression and enzymatic activities in hydroponic lettuce

**Figure 3:**
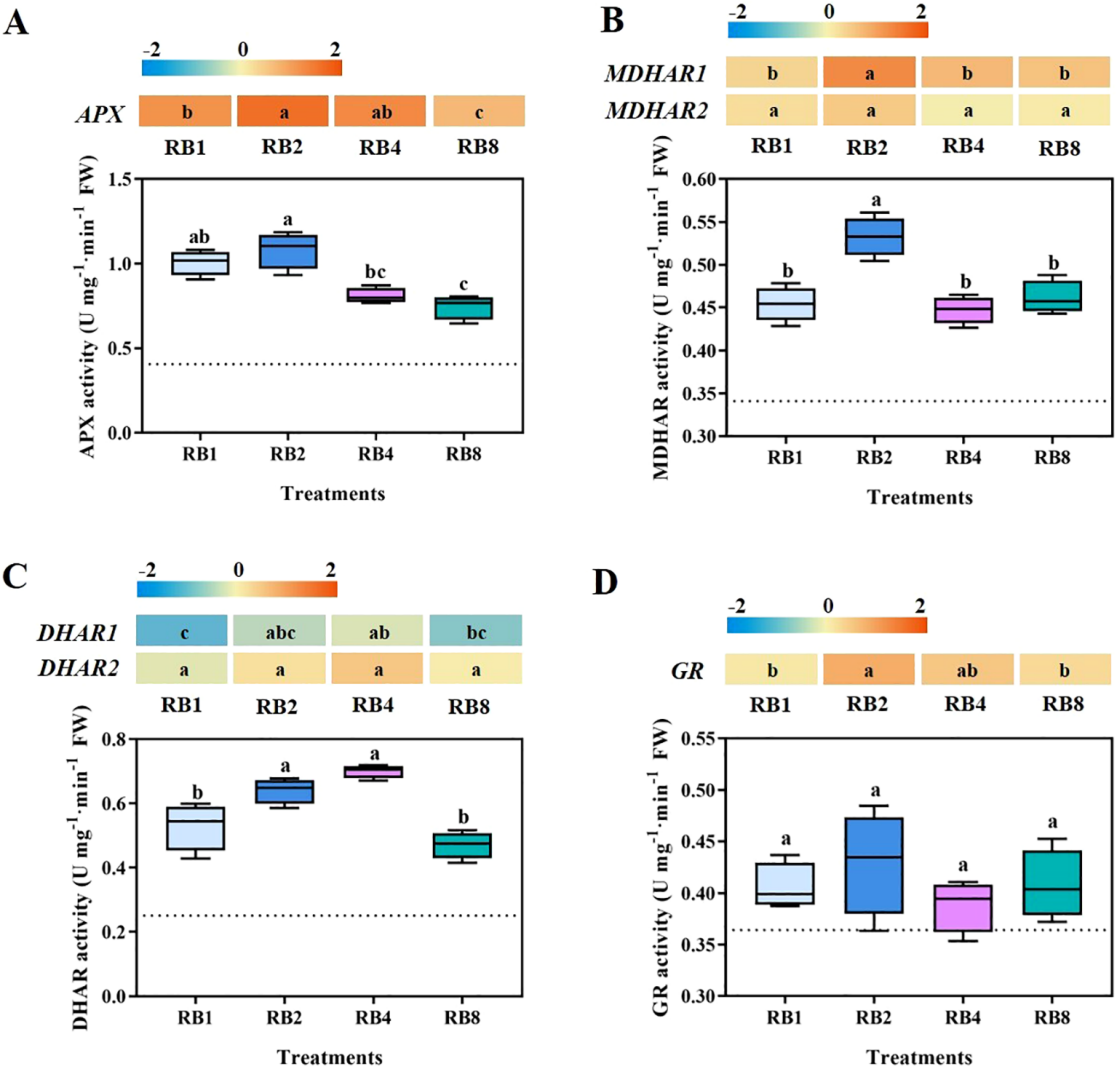
Key enzyme activities (including APX, MDHAR, DHAR, and GR) and expression of related genes (including *APX, MDHAR1, MDHAR2, DHAR1, DHAR2*, and *GR*) involved in AsA cycling of plants grown under different light treatments at the end-of-production (EOP) stage. RB1 (continuous illumination), RB2 (4 h light followed by 4 h dark and concluding with a final 4 h light), RB4 (three cycles of 2 h light/2 h dark plus a final 2 h light), and RB8 (seven cycles of 1 h light/1 h dark with a final 1 h light). The dotted line indicates the sampling before EOP irradiation. Different letters indicate significant differences using Duncan’s multiple range test (*p* < 0.05; n = 4). Gene expression analyses were performed with four biological replicates, each including three technical replicates.

The EOP light/dark alternation differentially regulated the expression and activity of key enzymes in the AsA cycle. For APX, gene expression levels ranked as RB2 > RB4 > RB1 > RB8. In contrast, APX enzyme activity was significantly higher in RB2 than in RB4 and RB8 but was comparable to that in RB1 (**Figure 3A**). Both *MDHAR1* and *MDHAR2* expression and enzymatic activity peaked under the RB2 treatment. Specifically, MDHAR activity in RB2 was significantly higher than in RB1, RB4, and RB8 (by 17.3%, 19.1%, and 15.4%, respectively; **Supplementary Table S1**), while no significant differences were observed among the latter three treatments (**Figure 3B**). *DHAR1* and *DHAR2* expression was highest in the RB4 treatment. Accordingly, DHAR activity was also elevated under RB4, showing no significant difference from RB2, but both were significantly higher than in RB1 and RB8 (**Figure 3C**). Finally, although *GR* gene expression varied significantly across treatments, no corresponding significant differences were detected in its enzyme activity (**Figure 3D**).

### 3.5 Effect of EOP light treatment under different light/dark alternating frequencies on soluble sugar content

**Figure 4:**
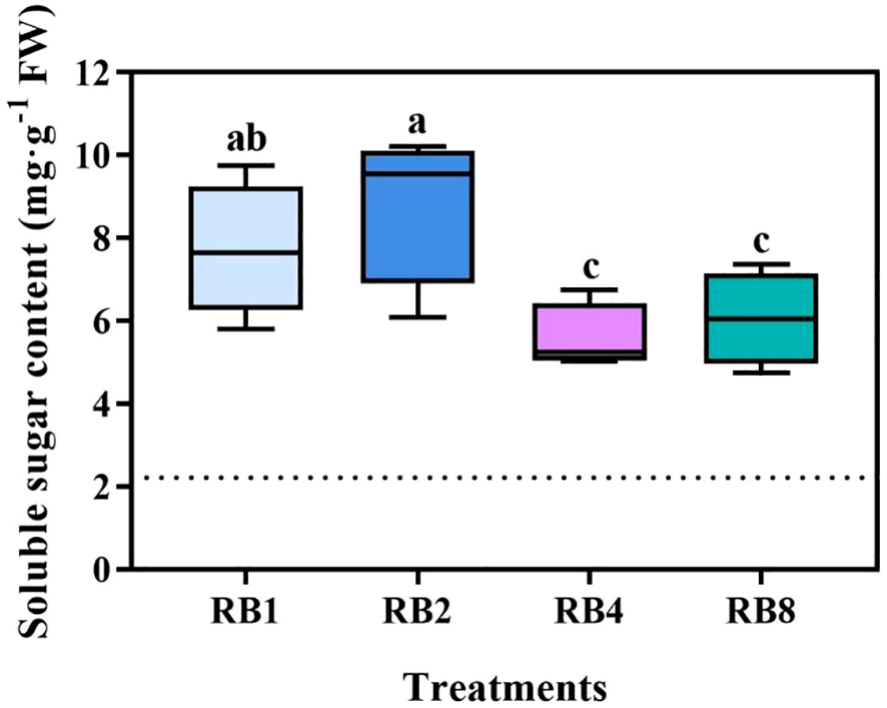
Soluble sugar content in lettuce leaves of plants grown under different light treatments at the end-of-production (EOP) stage: RB1 (continuous illumination), RB2 (4 h light followed by 4 h dark and concluding with a final 4 h light), RB4 (three cycles of 2 h light/2 h dark plus a final 2 h light), and RB8 (seven cycles of 1 h light/1 h dark with a final 1 h light). The dotted line indicates the sampling before EOP irradiation. Different letters indicate significant differences using Duncan’s multiple range test (*p* < 0.05; n = 4).

Different light/dark alternating treatments at the EOP stage had a significant effect on the soluble sugar content of hydroponic lettuce (**Figure 4**). Among the treatments, RB2 (8.9 mg·g^-1^ FW) resulted in the highest increase in soluble sugar, followed by RB1 (**Supplementary Table S2**). The soluble sugar content under RB2 was significantly higher than that under RB4 and RB8, with increases of 58.9% and 46.2% compared to RB4 and RB8, respectively.

### 3.6 Effect of EOP light treatment under different light/dark alternating frequencies on superoxide, hydrogen peroxide, and malondialdehyde accumulation in hydroponic lettuce

**Figure 5:**
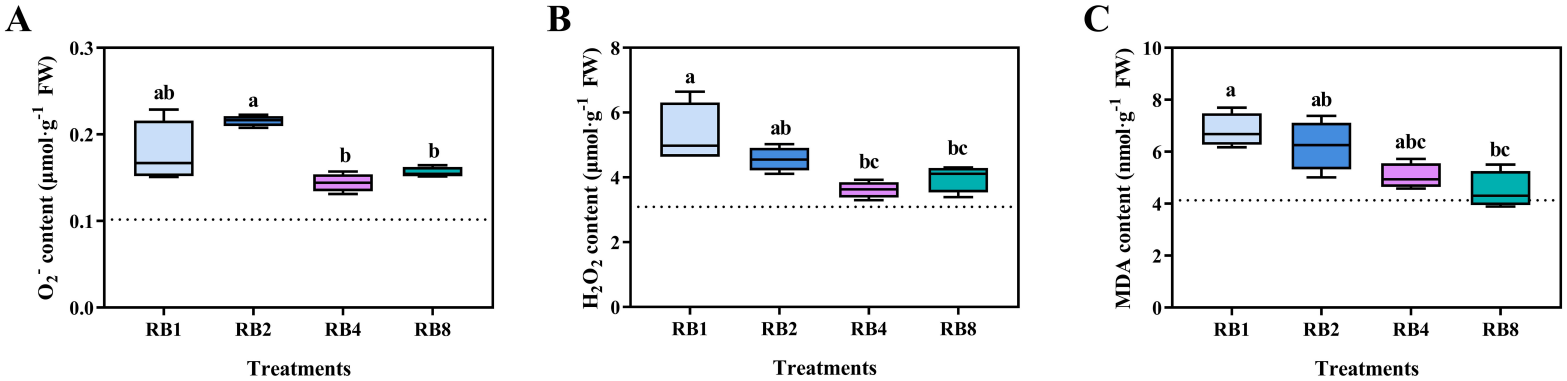
O_2_^-^**(A)**, H_2_O_2_**(B)**, and MDA **(C)** contents in lettuce leaves of plants grown under different light treatments at the end-of-production (EOP) stage: RB1 (continuous illumination), RB2 (4 h light followed by 4 h dark and concluding with a final 4 h light), RB4 (three cycles of 2 h light/2 h dark plus a final 2 h light), and RB8 (seven cycles of 1 h light/1 h dark with a final 1 h light). The dotted line indicates the sampling before EOP irradiation. Different letters indicate significant differences using Duncan’s multiple range test (*p* < 0.05; n = 4).

The contents of reactive oxygen species and MDA in response to different light/dark alternation treatments are shown in **Figure 5**. Regarding the O_2_^-^ content, there was no significant difference between RB1 and RB2, and RB4 and RB8 were significantly lower than RB2. For the H_2_O_2_ content, RB1 was significantly higher than RB4 and RB8, while there was no significant difference among RB2, RB4 and RB8. As the light/dark alternation frequency increased, MDA content in lettuce leaves gradually decreased (**Figure 5C**). The MDA content in RB1 was significantly higher than that in RB8, while there was no statistically significant difference in MDA content among the RB2, RB4, and RB8 treatments (**Supplementary Table S3**).

### 3.7 Correlation analysis

**Figure 6:**
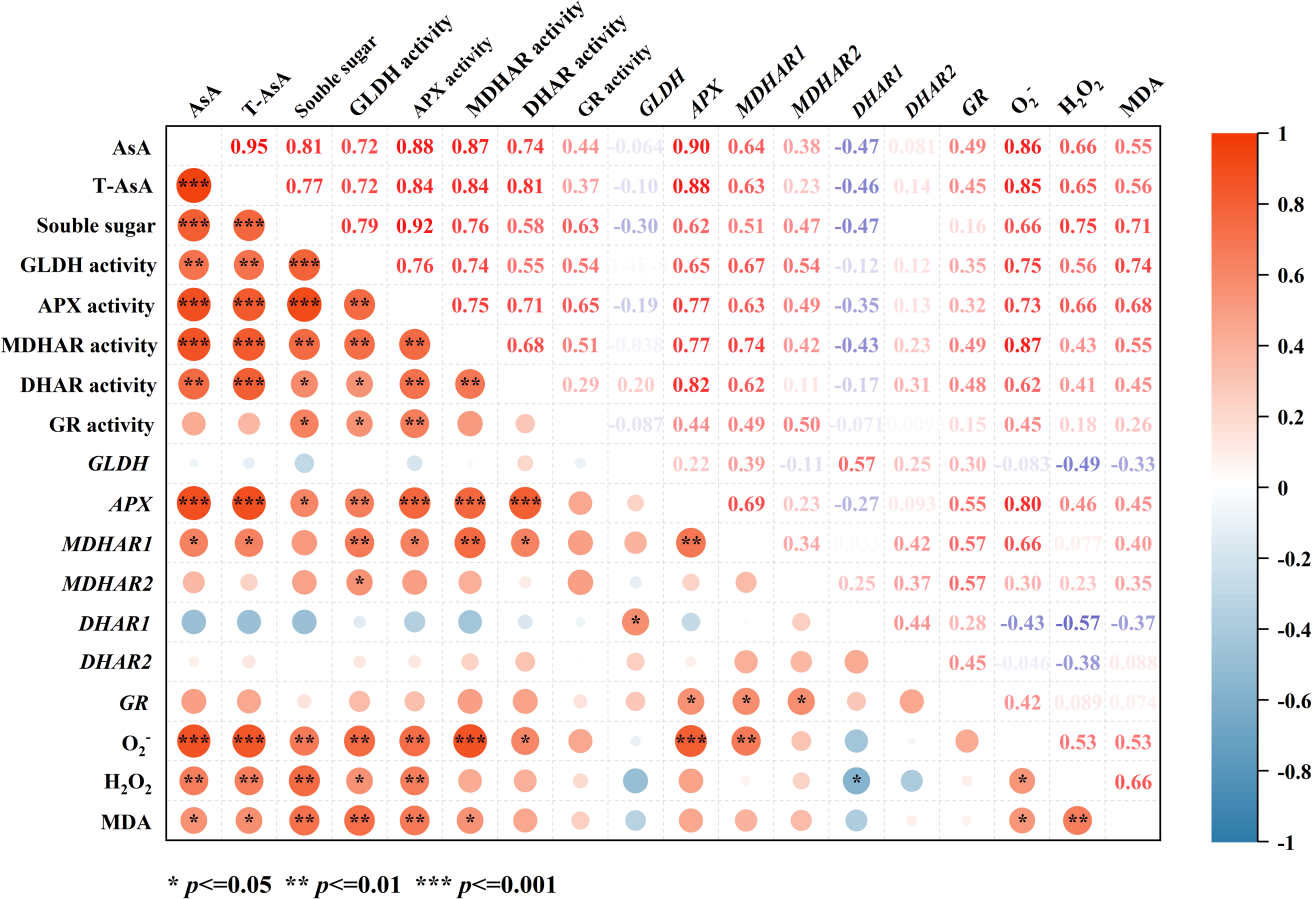
Correlation heatmap of the contents of AsA, T-AsA, soluble sugar, O_2_^-^, H_2_O_2_, MDA, as well as the activities and gene expression levels of GLDH, APX, DHAR, MDHAR, and GR in hydroponic lettuce under different light/dark alternating frequencies at the EOP light treatment. The color scale indicates the strength and direction of correlations (red: positive; blue: negative). Significance levels are denoted as *p < 0.05, **p < 0.01, ***p < 0.001.

Correlation analysis revealed that AsA and T-AsA showed highly significant positive correlations with GLDH, APX, MDHAR, and DHAR (*r* ≥ 0.72, *p* < 0.01, **Figure 6**). Their relationships with genes involved in synthesis and recycling enzymes were divergent: while strongly positively correlated with *APX* expression (*r* ≥ 0.88, *p* < 0.001), they showed no significant correlation with the expression levels of *MDHAR2* and *DHAR1*. Additionally, AsA and T-AsA were significantly positively correlated with ROS (O_2_^-^, H_2_O_2_) and the oxidative stress marker MDA. A highly significant positive correlation was also observed with soluble sugar content (*r* ≥ 0.77, *p* < 0.001).

### 3.8 Relationship between the duration of the final light period and AsA, T-AsA, soluble sugar, O_2_^-^, H_2_O_2_, and MDA contents

**Figure 7:**
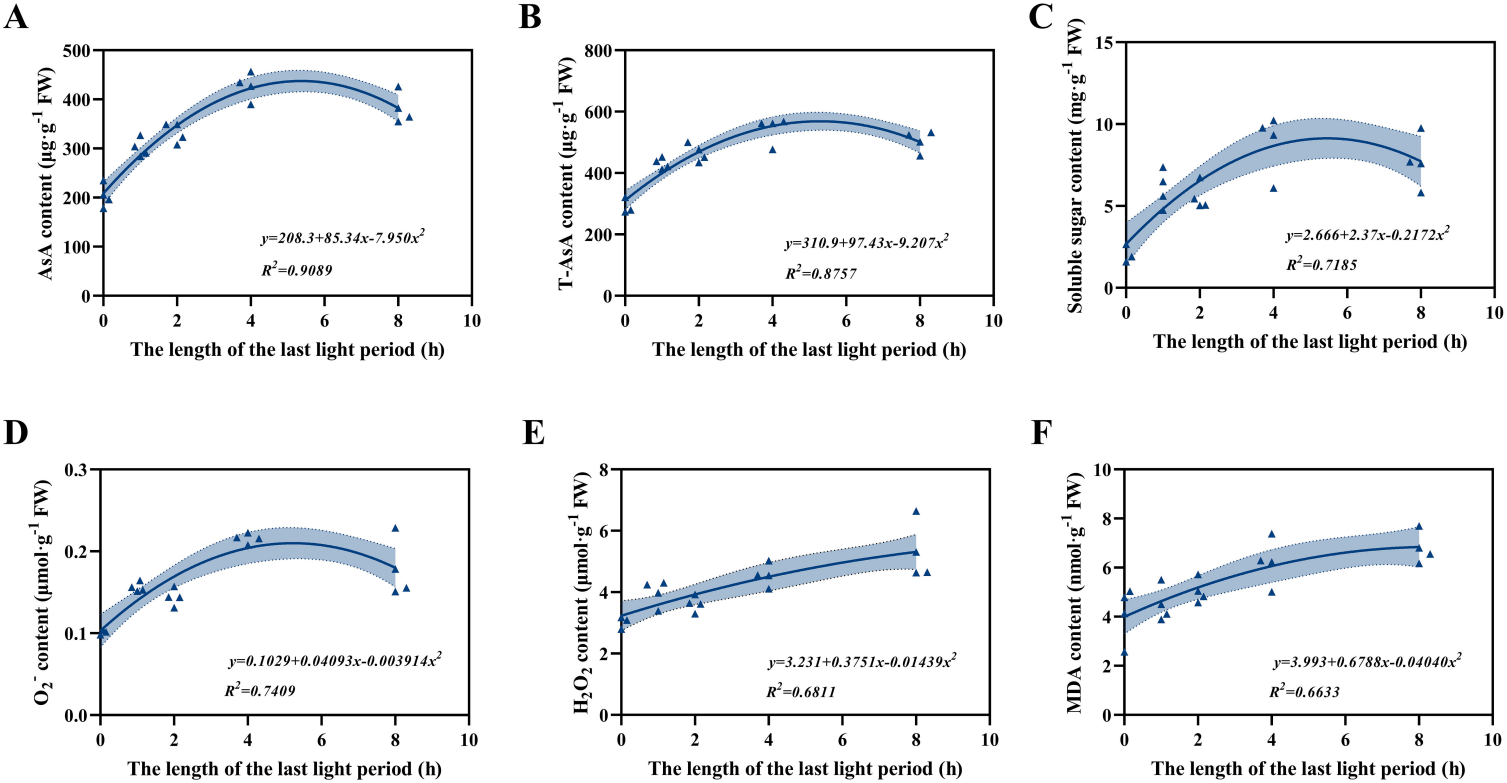
Fitting analysis of the relationships between the duration of the final light period and contents of AsA **(A)**, T-AsA **(B)**, soluble sugar **(C)**, O_2_^-^**(D)**, H_2_O_2_**(E)**, and MDA **(F)** in lettuce leaves. Each data point represents an individual biological replicate (n = 4). Regression equations and coefficients of determination (*R*^2^) are shown in each panel; shaded areas indicate 95% confidence intervals.

Fitting analysis revealed distinct curvilinear responses of lettuce physiological indices to the duration of the final light period (**Figure 7**). The contents of AsA, T-AsA, soluble sugar, and all increased initially and then decreased, showing obvious peak values at intermediate light durations. In contrast, H_2_O_2_ and MDA displayed continuous increases without obvious peaks, indicating a progressive accumulation of oxidative stress under prolonged illumination. These findings suggest that moderate light duration promotes antioxidant accumulation and sugar metabolism, whereas excessively long illumination enhances ROS generation and lipid peroxidation.

## 4 Discussion

Under high light conditions, both enzymatic activities and gene expression related to T-AsA biosynthesis and recycling are induced in leaves and fruits (Bartoli et al., 2006; Yabuta et al., 2007; Dowdle et al., 2007). This light-regulatory mechanism is believed to be mediated by photosynthesis (through plastoquinone status modulation) and respiration (Ntagkas et al., 2018). Phytochromes (red/far-red) and cryptochromes (blue) are known to modulate ROS homeostasis and activate antioxidant defense pathways (Wu et al., 2024; Chibani et al., 2025). Consistently, our previous research indicated that during EOP, increasing the proportion of blue light significantly enhances the activities of enzymes involved in AsA biosynthesis and recycling (Zhou et al., 2023). The size of the AsA pool, which is regulated by both biosynthesis and recycling pathways (Liu et al., 2023; Zha et al., 2020), is largely determined by the terminal enzyme in the D-mannose/L-galactose pathway, GLDH, a key enzyme for AsA biosynthesis (Bartoli et al., 2006; Zha et al., 2019a), alongside the crucial regeneration of AsA from its oxidized forms (MDHA and DHA). In this study, the RB2 treatment produced the highest AsA induction (increased by 11.8%, 28.5%, and 41.6% compared to RB1, RB4, and RB8, respectively), achieved through synchronized increases in *GLDH* expression/activity (**Figure 2**) and coordinated upregulation of the *APX*/*MDHAR*/*DHAR* system (**Figures 3A–C**). This aligns with previous findings: tobacco with suppressed *GLDH* expression showed reduced AsA content (Tabata et al., 2001), while *GLDH*-overexpressing plants exhibited expanded AsA pools (Liu et al., 2011); enhanced APX enzyme activity directly elevates AsA levels (Zha et al., 2019b), and overexpression of *MDHAR/DHAR* genes also significantly increases AsA content (Eltayeb et al., 2006; Chen et al., 2003; Eltayeb et al., 2007).

In *Arabidopsis*, increased AsA content inhibits *VTC2* transcription, which encodes GDP-L-galactose phosphorylase, thereby preventing excessive AsA accumulation through this negative feedback mechanism (Dowdle et al., 2007). Early evidence of this transcriptional regulation came from studies in tobacco, where exogenous AsA application significantly suppressed the expression of key biosynthetic enzyme genes such as *GMPase* and *GLDH* (Tabata et al., 2002). Our previous research has revealed that under continuous high light conditions, once AsA reaches a specific threshold, dual feedback inhibition is triggered on both biosynthetic genes (e.g., *GLDH*) and recycling genes (e.g., *APX, MDHAR*), forming a more complex regulatory network (Zhou et al., 2021). The RB2 protocol may partially overcome this limitation through light/dark alternation: AsA consumption during dark periods for ROS scavenging reduces cellular AsA concentration, thereby alleviating feedback inhibition. The subsequent light period then restores the activation of both biosynthetic and recycling pathways (**Figure 2**), creating a pulsatile accumulation pattern. Crucially, the 4 h photoperiod in RB2 provides optimal duration for light-induced gene expression without triggering feedback inhibition. Previous transcriptomic analyses demonstrate that light-regulated genes (e.g., *MDHAR, DHAR*) require sustained illumination (≥2 h) for substantial activation (Zhou et al., 2021). Shorter cycles (RB4/RB8) fail to provide sufficient induction time for gene expression, resulting in inadequate enzyme synthesis. Under severe stress conditions, cooperation between AsA and glutathione (GSH) becomes essential for enhanced AsA recycling (Tuzet et al., 2019). Although 8 h high light treatment induced ROS accumulation, no significant differences in GR activity were observed among treatments (**Supplementary Table S4**), suggesting ROS levels remained below the threshold required for significant activation of GSH-AsA cycling.

When plants acclimated to low light are abruptly exposed to high light, the energy input can exceed their photosynthetic capacity, leading to suppressed photochemical efficiency and excessive ROS generation in chloroplasts and mitochondria (Takahashi and Badger, 2011; Alter et al., 2012; Petrov et al., 2015). Moreover, blue light can enhance ROS formation under both normal and high light intensities (Chibani et al., 2025). In this study, significant increases in O_2_^-^ and H_2_O_2_ under both RB1 and the RB2 treatments confirmed that prolonged exposure (>4 h) to the specific light regimen (500 μmol·m^-^²·s^-1^; red: blue = 1:1) exceeded lettuce’s oxidative stress tolerance threshold and induced photooxidative stress (**Figure 5**). The imbalance between ROS production and scavenging exacerbated membrane lipid peroxidation, as reflected by elevated MDA content. Furthermore, fitting analyses revealed that prolonged final light duration promoted continuous accumulation of O_2_^-^, H_2_O_2_, and MDA (**Figure 7**), highlighting a positive correlation between oxidative stress and the duration of the final light exposure.

AsA plays a central role in protecting plants against oxidative damage by directly scavenging ROS and serving as a cofactor for antioxidant enzymes under stress (Foyer and Noctor, 2011; Raja et al., 2017; Min et al., 2021; Vidal-Meireles et al., 2017). In this study, correlation analysis revealed that AsA and T-AsA levels were positively correlated with ROS (O_2_^-^, H_2_O_2_) and MDA, showing a particularly strong correlation with O_2_^-^ (**Figure 6**). This positive correlation likely reflects a stress-response mechanism where ROS accumulation under high light acts as a signal to upregulate the AsA system. Consequently, as the light/dark alternation frequency increased, leading to a reduction in oxidative stress (**Figure 5**), the contents of AsA and T-AsA also exhibited a corresponding decline (**Table 3**), confirming the tight coupling between ROS signaling and AsA metabolism. The lower levels of ROS and MDA observed under higher alternation frequencies can be attributed to two key factors: (1) the dissipation of excess light energy and the utilization of photosynthetic products during the dark phase, which mitigates over-reduction of the electron transport chain, and (2) more effective interruption of continuous photooxidative damage with more frequent light/dark cycles, thereby minimizing the accumulation of ROS and subsequent membrane peroxidation.

Carbohydrates, the primary assimilates of photosynthesis, provide carbon skeletons and energy for organic compound synthesis (Rolland et al., 2006). High light may enhance AsA biosynthesis by promoting carbohydrate accumulation (Zhou et al., 2021). Nishikawa et al. (2005) showed that increasing the content of sucrose upregulate the expression of genes involved in AsA biosynthesis pathway and delay the loss of AsA in broccoli. In the present study, correlation analysis revealed that AsA and T-AsA exhibited significant positive correlations with both soluble sugar content and the activities of key antioxidant enzymes (**Figure 6**), further supporting the link between carbon metabolism and antioxidant capacity.

In summary, this study demonstrates that EOP light/dark alternation regulates AsA metabolism in lettuce. The RB2 treatment protocol optimally balanced the relationship between ROS levels and the expression and activity of enzymes involved in both AsA biosynthesis and regeneration, thereby achieving maximal AsA accumulation. This study further confirmed significant correlations between AsA and oxidative stress products (O_2_^-^, H_2_O_2_, MDA) as well as soluble sugars. Although the short-term light treatments showed no significant effects on growth traits, the RB2 protocol presents a promising, energy-saving strategy for enhancing the nutritional quality of lettuce under EOP conditions.

A potential limitation of this study is that gene transcription and metabolic processes fluctuate with circadian rhythms. Consequently, the observed treatment differences may stem not only from the direct effects of the light environment (intensity and spectral composition, and alternation patterns) but also from the regulatory influence of the circadian clock. To address this, future studies will include a continuous dark control group and implement dynamic sampling. Specifically, samples from this control will be collected at 8, 12, 14, and 15 hours after the initiation of light exposure in the treatment groups. This design will help distinguish whether the observed phenomena are direct light effects or circadian-driven, thereby clarifying the regulatory roles of the circadian clock versus light/dark alternation patterns in AsA metabolism and related gene expression. In addition, to clarify the independent contribution of the light treatment itself (500 μmol·m^-^²·s^-1^; red: blue = 1:1) to ascorbic acid (AsA) accumulation, the T0 sampling design will be optimized in subsequent studies: samples will be collected after 8 h of continuous light under the initial growth conditions (200 μmol·m^-^²·s^-1^; red: blue = 3:1), and this data will be used as the new T0 baseline value for direct comparison with the RB1 treatment group (500 μmol·m^-^²·s^-1^; red: blue = 1:1, 8 h of continuous light).

## 5 Conclusions

Overall, this study demonstrates that EOP application of high light with a high blue light ratio, combined with light/dark alternation, enables effective regulation of AsA metabolism in hydroponic lettuce. Among the tested protocols, the RB2 treatment (4 h light/4 h dark/4 h light) optimally balanced ROS levels with the expression and activity of key enzymes in both AsA biosynthesis and regeneration, resulting in maximal AsA accumulation. Although the short-term light treatments showed no significant effects on growth traits, the RB2 protocol represents a practical and energy-efficient strategy for enhancing the nutritional quality of lettuce in controlled environment agriculture.

## Data Availability

All data generated or analyzed during this study are included in this published article and its Supplementary Information files.

## Supplementary material

Supplementary Table 1 GLDH activity and Key enzymes involved in AsA cycling activities in lettuce leaves of plants grown under different light treatments at the end-of-production (EOP) stage: T0 (pre-treatment baseline), RB1 (continuous illumination), RB2 (4 h light followed by 4 h dark and concluding with a final 4 h light), RB4 (three cycles of 2 h light/2 h dark plus a final 2 h light), and RB8 (seven cycles of 1 h light/1 h dark with a final 1 h light). Values represent the means of four replicates ± SD. Different letters indicate significant differences using Duncan’s multiple range test (*p* < 0.05; n = 4).

Supplementary Table 2 Soluble sugar content in lettuce leaves of plants grown under different light treatments at the end-of-production (EOP) stage: T0 (pre-treatment baseline), RB1 (continuous illumination), RB2 (4 h light followed by 4 h dark and concluding with a final 4 h light), RB4 (three cycles of 2 h light/2 h dark plus a final 2 h light), and RB8 (seven cycles of 1 h light/1 h dark with a final 1 h light). Values represent the means of four replicates ± SD. Different letters indicate significant differences using Duncan’s multiple range test (*p* < 0.05; n = 4).

Supplementary Table 3 O_2_^-^, H_2_O_2_ and MDA contents in lettuce leaves of plants grown under different light treatments at the end-of-production (EOP) stage: T0 (pre-treatment baseline), RB1 (continuous illumination), RB2 (4 h light followed by 4 h dark and concluding with a final 4 h light), RB4 (three cycles of 2 h light/2 h dark plus a final 2 h light), and RB8 (seven cycles of 1 h light/1 h dark with a final 1 h light). Values represent the means of four replicates ± SD. Different letters indicate significant differences using Duncan’s multiple range test (*p* < 0.05; n = 4).

Supplementary Table 4 Gene expression of *GLDH* and Key enzymes in AsA cycling in lettuce leaves of plants grown under different light treatments at the end-of-production (EOP) stage: T0 (pre-treatment baseline), RB1 (continuous illumination), RB2 (4 h light followed by 4 h dark and concluding with a final 4 h light), RB4 (three cycles of 2 h light/2 h dark plus a final 2 h light), and RB8 (seven cycles of 1 h light/1 h dark with a final 1 h light). Values represent the means of four replicates ± SD. Different letters indicate significant differences using Duncan’s multiple range test (*p* < 0.05; n = 4).

Supplementary Figure 1 Cultivation status of lettuce plants after 16 days of hydroponics under artificial light.
