# Correction: Effect of end-of-production light treatment under different light/dark alternating frequencies on ascorbic acid accumulation and metabolism of hydroponic lettuce

**DOI:** 10.3389/fpls.2025.1764279

**Published:** 2025-12-19

**Authors:** Frontiers Production Office

**Affiliations:** Frontiers Media SA, Lausanne, Switzerland

**Keywords:** ascorbic acid (AsA), light/dark alternating, end-of-production (EOP), reactive oxygen species (ROS), hydroponic lettuce

There was a mistake in the citations for **Figures 2–7** as published. The order of the figures was updated; however the citations were not updated and some citations were not put into parentheses. “EOP light/dark alternation differentially regulated GLDH in lettuce leaves **Figure 2**” has been updated to “EOP light/dark alternation differentially regulated GLDH in lettuce leaves (**Figure 2**)”. “In contrast, APX enzyme activity was significantly higher in RB2 than in RB4 and RB8 but was comparable to that in RB1 (**Figure 2A**)” has been updated to “In contrast, APX enzyme activity was significantly higher in RB2 than in RB4 and RB8 but was comparable to that in RB1 (**Figure 3A**)”. “while no significant differences were observed among the latter three treatments (**Figure 2B**)” has been updated to “while no significant differences were observed among the latter three treatments (**Figure 3B**)”. “but both were significantly higher than in RB1 and RB8 (**Figure 2C**)” has been updated to “but both were significantly higher than in RB1 and RB8 (**Figure 3C**)”. “Finally, although GR gene expression varied significantly across treatments, no corresponding significant differences were detected in its enzyme activity (**Figure 2D**)” has been updated to “Finally, although GR gene expression varied significantly across treatments, no corresponding significant differences were detected in its enzyme activity (**Figure 3D**)”. “Different light/dark alternating treatments at the EOP stage had a significant effect on the soluble sugar content of hydroponic lettuce (**Figure 3**)” has been updated to “Different light/dark alternating treatments at the EOP stage had a significant effect on the soluble sugar content of hydroponic lettuce (**Figure 4**)”. “followed by RB1 Supplementary Table S2” has been updated to “followed by RB1 (**Supplementary Table S2**)”. “The contents of reactive oxygen species and MDA in response to different light/dark alternation treatments are shown in **Figure 4**” has been updated to “The contents of reactive oxygen species and MDA in response to different light/dark alternation treatments are shown in **Figure 5**”. “As the light/dark alternation frequency increased, MDA content in lettuce leaves gradually decreased (**Figure 4C**)” has been updated to “As the light/dark alternation frequency increased, MDA content in lettuce leaves gradually decreased (**Figure 5C**)”. “and RB8 treatments **Supplementary Table S3**” has been updated to “and RB8 treatments (**Supplementary Table S3**)”. “Correlation analysis revealed that AsA and T-AsA showed highly significant positive correlations with GLDH, APX, MDHAR, and DHAR (r ≥ 0.72, p < 0.01) **Figure 6**” has been updated to “Correlation analysis revealed that AsA and T-AsA showed highly significant positive correlations with GLDH, APX, MDHAR, and DHAR (r ≥ 0.72, p < 0.01 **Figure 6**)”. “Fitting analysis revealed distinct curvilinear responses of lettuce physiological indices to the duration of the final light period (**Figure 5**)” has been updated to “Fitting analysis revealed distinct curvilinear responses of lettuce physiological indices to the duration of the final light period (**Figure 7**)”. “In this study, the RB2 treatment produced the highest AsA induction (increased by 11.8%, 28.5%, and 41.6% compared to RB1, RB4, and RB8, respectively), achieved through synchronized increases in GLDH expression/activity (**Figure 6**) and coordinated upregulation of the APX/MDHAR/DHAR system(**Figures 2A–C**)” has been updated to “In this study, the RB2 treatment produced the highest AsA induction (increased by 11.8%, 28.5%, and 41.6% compared to RB1, RB4, and RB8, respectively), achieved through synchronized increases in GLDH expression/activity (**Figure 2**) and coordinated upregulation of the APX/MDHAR/DHAR system (**Figures 3A–C**)”. “The subsequent light period then restores the activation of both biosynthetic and recycling pathways (**Figure 6**)” has been updated to “The subsequent light period then restores the activation of both biosynthetic and recycling pathways (**Figure 2**)”. “exceeded lettuce’s oxidative stress tolerance threshold and induced photooxidative stress (**Figure 4**)” has been updated to “exceeded lettuce’s oxidative stress tolerance threshold and induced photooxidative stress (**Figure 5**)”. “continuous accumulation of O_2_-, H_2_O_2_, and MDA (**Figure 5**)” has been updated to “continuous accumulation of O_2_-, H_2_O_2_, and MDA (**Figure 7**)”. “showing a particularly strong correlation with O_2_- (**Figure 7**)” has been updated to “showing a particularly strong correlation with O_2_- (**Figure 6**)”. “Consequently, as the light/dark alternation frequency increased, leading to a reduction in oxidative stress (**Figure 4**)” has been updated to “Consequently, as the light/dark alternation frequency increased, leading to a reduction in oxidative stress (**Figure 5**)”. “significant positive correlations with both soluble sugar content and the activities of key antioxidant enzymes (**Figure 7**)” has been updated to “significant positive correlations with both soluble sugar content and the activities of key antioxidant enzymes (**Figure 6**)”.

**Figure 2:**
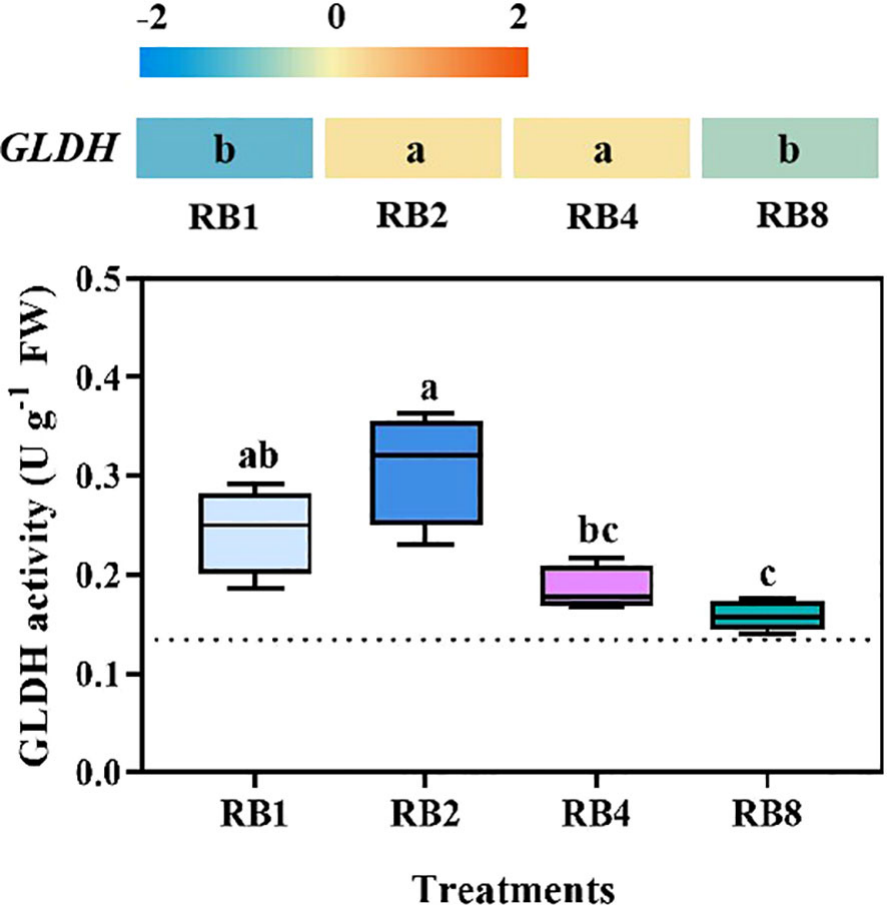
GLDH expression and GLDH activity in lettuce leaves of plants grown under different light treatments at the end-of-production (EOP) stage:RB1 (continuous illumination),RB2(4h light followed by 4h dark and concluding with a final 4h light),RB4 (three cycles of 2h light/2h dark plus a final 2h light), and RB8 (seven cycles of 1h light/1h dark with a final 1h light). The dotted line indicates the sampling before EOP irradiation. Different letters indicate significant differences using Duncan’s multiple range test (p < 0.05;n=4). Gene expression analyses were performed with four biological replicates, each including three technical replicates.

The image for **Figure 2** is incorrect as published. The figure image of **Figure 4** was duplicated to **Figure 2**. The correct version of **Figure 2** can be seen below.

The original version of this article has been updated.

