# Supplementary material for: Effect of end-of-production light treatment under different light/dark alternating frequencies on ascorbic acid accumulation and metabolism of hydroponic lettuce
Source: Front Plant Sci. 2025 Dec 3;16:1681893. doi: 10.3389/fpls.2025.1681893 (PMC12708927; doi:10.3389/fpls.2025.1681893)
Supplement: Supplementary Table 1 — GLDH activity and Key enzymes involved in AsA cycling activities in lettuce leaves of plants grown under different light treatments at the end-of-production (EOP) stage: T0 (pre-treatment baseline), RB1 (continuous illumination), RB2 (4 h light followed by 4 h dark and concluding with a final 4 h light), RB4 (three cycles of 2 h light/2 h dark plus a final 2 h light), and RB8 (seven cycles of 1 h light/1 h dark with a final 1 h light). Values represent the means of four replicates ± SD. Different letters indicate significant differences using Duncan’s multiple range test (p < 0.05; n = 4). [file DataSheet1.pdf]

Supplementary Figure

Table S1. GLDH activity and Key enzymes involved in AsA cycling activities in lettuce leaves of plants grown under different light treatments at the end-of-production (EOP) stage: T0 (pre-treatment baseline), RB1 (continuous illumination), RB2 (4 h light followed by 4 h dark and concluding with a final 4 h light), RB4 (three cycles of 2 h light/2 h dark plus a final 2 h light), and RB8 (seven cycles of 1 h light/1 h dark with a final 1 h light). Values represent the means of four replicates ± SD. Different letters indicate significant differences using Duncan's multiple range test ($p < 0.05$; n = 4).

Treatment	GLDH (U $g^{-1}\cdot min^{-1}$ FW)	APX (U $mg^{-1}\cdot min^{-1}$ FW)	DHAR (U $mg^{-1}\cdot min^{-1}$ FW)	MDHAR (U $mg^{-1}\cdot min^{-1}$ FW)	GR (U $mg^{-1}\cdot min^{-1}$ FW)
T0	0.13±0.02 c	0.41±0.17 d	0.25±0.04 c	0.34±0.04 c	0.36±0.03 a
RB1	0.24±0.05 ab	1.01±0.09 ab	0.53±0.09 b	0.45±0.02 b	0.41±0.03 a
RB2	0.31±0.07 a	1.08±0.13 a	0.64±0.05 a	0.53±0.03 a	0.43±0.06 a
RB4	0.18±0.03 bc	0.81±0.05 bc	0.70±0.03 a	0.45±0.02 b	0.39±0.03 a
RB8	0.16±0.02 c	0.75±0.09 c	0.47±0.05 b	0.46±0.02 b	0.41±0.04 a

Table S2. Soluble sugar content in lettuce leaves of plants grown under different light treatments at the end-of-production (EOP) stage: T0 (pre-treatment baseline), RB1 (continuous illumination), RB2 (4 h light followed by 4 h dark and concluding with a final 4 h light), RB4 (three cycles of 2 h light/2 h dark plus a final 2 h light), and RB8 (seven cycles of 1 h light/1 h dark with a final 1 h light). Values represent the means of four replicates ± SD. Different letters indicate significant differences using Duncan's multiple range test ($p < 0.05$; n = 4).

Treatment	Soluble sugar content ($mg\cdot g^{-1}$ FW)
T0	2.22±0.55 c
RB1	7.72±1.61 ab
RB2	8.85±1.88 a
RB4	5.57±0.81 b
RB8	6.06±1.13 b

Table S3. O_2^-, H_2O_2 and MDA contents in lettuce leaves of plants grown under different light treatments at the end-of-production (EOP) stage: T0 (pre-treatment baseline), RB1 (continuous illumination), RB2 (4 h light followed by 4 h dark and concluding with a final 4 h light), RB4 (three cycles of 2 h light/2 h dark plus a final 2 h light), and RB8 (seven cycles of 1 h light/1 h dark with a

final 1 h light). Values represent the means of four replicates ± SD. Different letters indicate significant differences using Duncan's multiple range test (p < 0.05; n = 4).

Treatment	O_2^- (μmol·g^{-1} FW)	H_2O_2 (μmol·g^{-1} FW)	MDA (nmol·g^{-1} FW)
T0	0.10±0.00 c	3.09±0.26 c	4.13±1.36 c
RB1	0.18±0.04 ab	5.31±1.15 a	6.81±0.79 a
RB2	0.22±0.01 a	4.56±0.46 ab	6.23±1.19 ab
RB4	0.14±0.01 b	3.62±0.31 bc	5.04±0.60 abc
RB8	0.16±0.01 b	3.98±0.51 bc	4.50±0.88 bc

Table S4. Gene expression of *GLDH* and Key enzymes in AsA cycling in lettuce leaves of plants grown under different light treatments at the end-of-production (EOP) stage: T0 (pre-treatment baseline), RB1 (continuous illumination), RB2 (4 h light followed by 4 h dark and concluding with a final 4 h light), RB4 (three cycles of 2 h light/2 h dark plus a final 2 h light), and RB8 (seven cycles of 1 h light/1 h dark with a final 1 h light). Values represent the means of four replicates ± SD. Different letters indicate significant differences using Duncan's multiple range test (p < 0.05; n = 4).

Treatment	*GLDH*	*APX*	*MDHAR1*	*MDHAR2*	*DHAR1*	*DHAR2*	*GR*
T0	1.10±0.20 a	1.01±0.18 c	1.06±0.22 b	1.07±0.25 a	1.07±0.33 a	1.03±0.32 a	1.05±0.38 b
RB1	0.50±0.00 b	2.28±0.26 b	1.33±0.17 b	1.23±0.30 a	0.47±0.08 c	0.92±0.14 a	1.12±0.16 b
RB2	1.23±0.10 a	3.10±0.38 a	2.53±0.27 a	1.48±0.45 a	0.77±0.23 abc	1.18±0.56 a	1.88±0.56 a
RB4	1.22±0.14 a	2.45±0.47 ab	1.68±0.29 b	1.06±0.44 a	0.90±0.19 ab	1.47±0.27 a	1.54±0.61 ab
RB8	0.74±0.13 b	1.61±0.25 c	1.55±0.59 b	1.10±0.23 a	0.59±0.13 bc	1.06±0.12 a	1.27±0.58 b

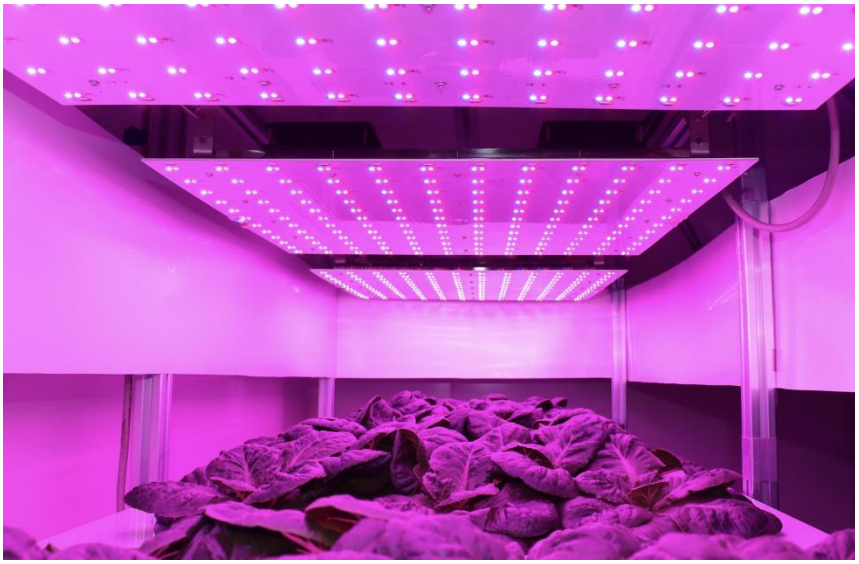

Figure S1. Cultivation status of lettuce plants after 16 days of hydroponics under artificial light.
